# Junior Doctor's Ideas, Concerns and Expectations About Electroconvulsive Therapy: An Educational Quality Improvement Project

**DOI:** 10.1192/bjo.2024.347

**Published:** 2024-08-01

**Authors:** Victoria Selwyn, Julian Navanathan, Rebecca Howard, Yzobelle Barcelos

**Affiliations:** 1 East and North Hertfordshire NHS Trust, Hertfordshire, United Kingdom; 2 West Hertfordshire NHS Trust, Hertfordshire, United Kingdom; 3 Princess Alexandra NHS Trust, Harlow, Essex, United Kingdom

## Abstract

**Aims:**

There remains stigma surrounding electroconvulsive therapy (ECT) amongst junior doctors, as well as gaps in knowledge, recent studies have shown. The aim of this study is to reduce stigma and negative biases towards ECT among junior doctors in Hertfordshire.

This research strives to improve clinical knowledge regarding ECT amongst the same population of junior doctors.

After highlighting stigma and gaps in clinical knowledge amongst junior doctors, we aimed to implement an educational intervention to reduce these and assess the impact it made.

**Methods:**

Over 80 doctors ranging from foundation year 1 doctors to consultants attended a weekly academic teaching for doctors working in Psychiatry. A 50-minute slot was set aside for a teaching session on ECT.

This included a pre- and post-teaching anonymous questionnaire, with open and closed questions, asking junior doctors about their previous exposure to ECT, and asking them to list three words they associated with ECT.

The teaching session included: what ECT is, indications, side effects, a short video explaining the procedure, an open discussion about stigma and ECT, a brief overview about the future of neuromodulation, and a consultant psychiatrist who is part of the ECT team talking through the before, during, after, and answering questions from the participants.

**Results:**

31 participants answered the pre-intervention questionnaire. Of the 31 respondents, 70% reported learning about ECT during medical school. However, 40% reported little teaching and only 13% had observed ECT. From thematic analysis of free text responses, 54% of respondents expressed detailed understanding of ECT, with 71% agreeing that ECT is a humane treatment. 80% expressed that ECT should be part of NICE guidelines. 50% of respondents conveyed that stigmatised portrayals of ECT in popular culture have influenced their negative opinion of ECT.

Of the 10 responses to the post-teaching questionnaire, 100% agreed that ECT is a humane treatment and that ECT should be part of NICE guidelines for treatment of severe/treatment-resistant depression. From thematic analysis, when asked to name 3 words they associated with ECT, 60% of participants described ECT as effective or successful and 40% described ECT as safe. 72% of the words used were positive descriptors.

**Conclusion:**

ECT is not covered thoroughly during medical school. Before this teaching, about half of the trainees expressed a negative opinion of ECT due to popular culture. Post-teaching, positive opinions had increased, and more trainees (100%) agreed that ECT is a humane treatment and should be part of NICE guidelines.

